# A city-year dataset of residual-based tail physical climate risk for Chinese cities, 1973 to 2024

**DOI:** 10.1016/j.dib.2026.113032

**Published:** 2026-06-30

**Authors:** ZheYu Guo, Han Sun, Hyoung-Goo Kang, XinYu Zhang

**Affiliations:** a Department of Computer Science and Financial Engineering, Graduate School, Hanyang University, Engineering Center Main Building, Room 503, 222 Wangsimni-ro, Seongdong-gu, Seoul, 04763, Republic of Korea; b Department of Finance, Hanyang University Business School, Room 706, 222 Wangsimni-ro, Seongdong-gu, Seoul, 04763, Republic of Korea

**Keywords:** Tail climate risk, Residual-based weather measure, City-year climate dataset, Extreme weather frequency, China, Urban climate exposure

## Abstract

This article describes a city-year dataset of residual-based tail physical climate risk for Chinese cities from 1973 to 2024. The released panel covers 368 prefecture-level cities and 19,136 city-year observations built from 6981,696 harmonized city-day records. The dataset is constructed from harmonized daily precipitation and temperature records and provides standardized annual indices of distributional tail risk for precipitation and temperature at the city level. For each city, the daily series are reduced to seasonal residuals by removing a low-order Fourier climatology in day of year, with no time trend or calendar fixed effects subtracted. Tail exposure is then accumulated from these residuals as annual exceedance intensities over a grid of city-specific quantile supports, standardized across city-years, and combined into composite precipitation, temperature, and overall city-level indices. The novelty of the dataset is that extremity is measured from city-specific seasonal residuals and defined relative to each city’s own empirical distribution rather than by common national or absolute thresholds, which makes the tail measures comparable across the heterogeneous climate regimes of China. The released files include the standardized composite tail-risk indices for precipitation, temperature, and the overall city level, geographic identifiers, a harmonized city-day weather panel, a city identifier crosswalk, construction code, a variable dictionary, and a README file. The dataset can be reused in climate finance, urban vulnerability, regional adaptation, environmental policy, and location-based exposure mapping.

Specifications Table

**Table:** 

Subject	Earth & Environmental Sciences
Specific subject area	City-level physical climate risk measurement from daily weather data
Type of data	Table; panel dataset; metadata file; code file; crosswalk file Raw; processed; harmonized; analyzed
Data collection	Daily city-level precipitation and temperature records for China were compiled from annual raw files, standardized across years, harmonized by city identifier, merged with geographic information, and processed into city-day and city-year panels. Each city-variable series was then reduced to a seasonal residual with a low-order Fourier climatology in day of year, with no time trend or calendar fixed effect removed, before annual exceedance intensities were accumulated over a grid of city-specific quantile supports and standardized into the released composite tail-risk indices. The underlying records are daily land-surface station observations from the Global Historical Climatology Network-Daily; precipitation is measured in millimeters and temperature in degrees Celsius, with the daily maximum and minimum temperature series used for the heat and cold tail measures. Stations are matched to prefecture-level cities under a fixed within-or-nearest-boundary rule, with multiple stations combined into a single city-day series, producing a balanced panel of 18,972 valid daily observations per city with no missing days imputed.
Data source location	China. Raw daily weather records are from the Global Historical Climatology Network-Daily archive (U.S. National Centers for Environmental Information); each released city carries its longitude and latitude. The dataset was assembled at Hanyang University, Seoul, Republic of Korea, and is archived on Zenodo (https://doi.org/10.5281/zenodo.19436740).
Data accessibility	Repository name: Zenodo Data identification number: https://doi.org/10.5281/zenodo.19436740 Direct URL to data: https://doi.org/10.5281/zenodo.19436740 Instructions for accessing these data: The repository is publicly accessible through the DOI link and contains the city-year panel, harmonized city-day panel, city identifier crosswalk, variable dictionary, construction code, and README file.
Related research article	none

## 1 Value of the Data

• The dataset provides a balanced city-year panel for 368 Chinese cities from 1973 to 2024 and measures tail physical climate exposure from residual daily weather processes rather than from annual averages or raw weather levels.
• The released composite indices let users separate precipitation-related and temperature-related tail exposure and compare an overall city-level tail-risk measure across locations and over time.
• The released files include both the final city-year panel and the harmonized city-day weather panel, which supports direct reuse in projects requiring annual indicators or alternative reconstructions from daily observations.
• The dataset can be merged with city-level socioeconomic, environmental, infrastructure, policy, or firm-location data for applications in climate finance, urban vulnerability, adaptation planning, regional analysis, and environmental management.
• Public access to the panel data, identifier crosswalk, variable dictionary, construction code, and README file reduces data preparation costs and facilitates transparent reuse, extension, and validation by other researchers.

## 2 Background

**Fig. 1:**
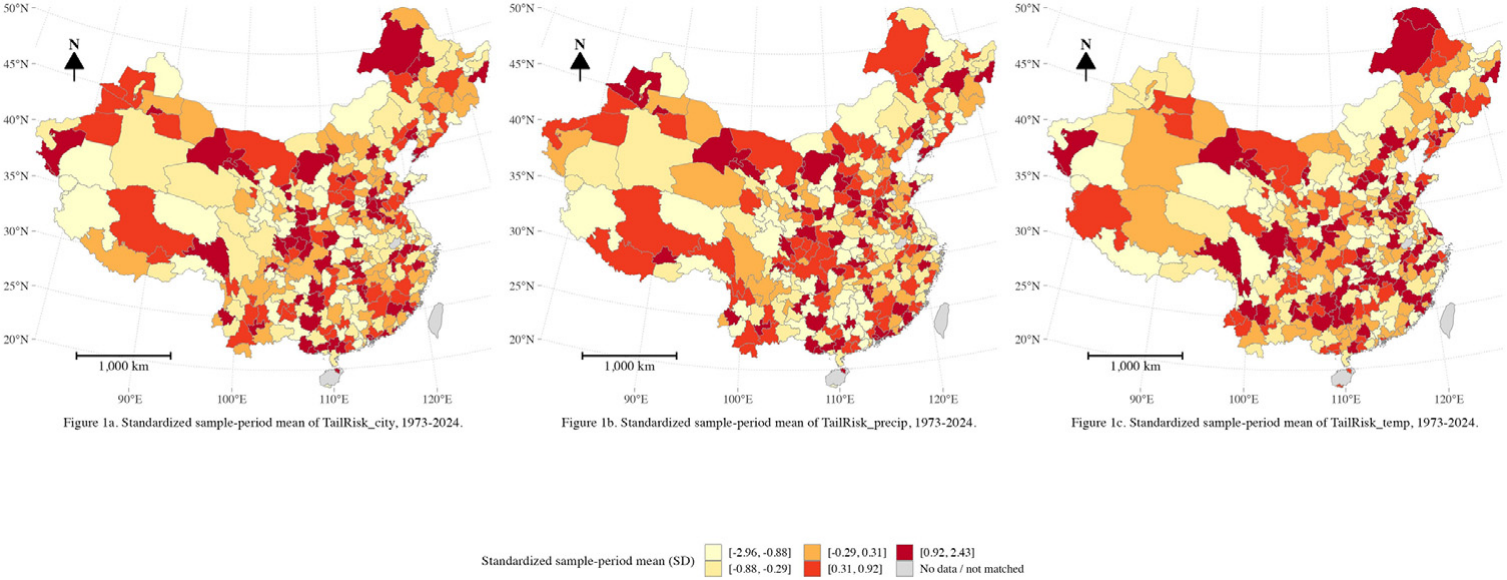
Spatial distribution of average released tail-risk indicators.

NOTE. Panel A reports quintile groups of the standardized sample-period mean of TailRisk_city. Panel B reports quintile groups of the standardized sample-period mean of TailRisk_precip. Panel C reports quintile groups of the standardized sample-period mean of TailRisk_temp. Each panel maps the 1973–2024 city mean of the released standardized index, expressed in cross-city standard-deviation units. The maps are based on cities successfully linked to the city map layer through the identifier matching procedure.

**Fig. 2:**
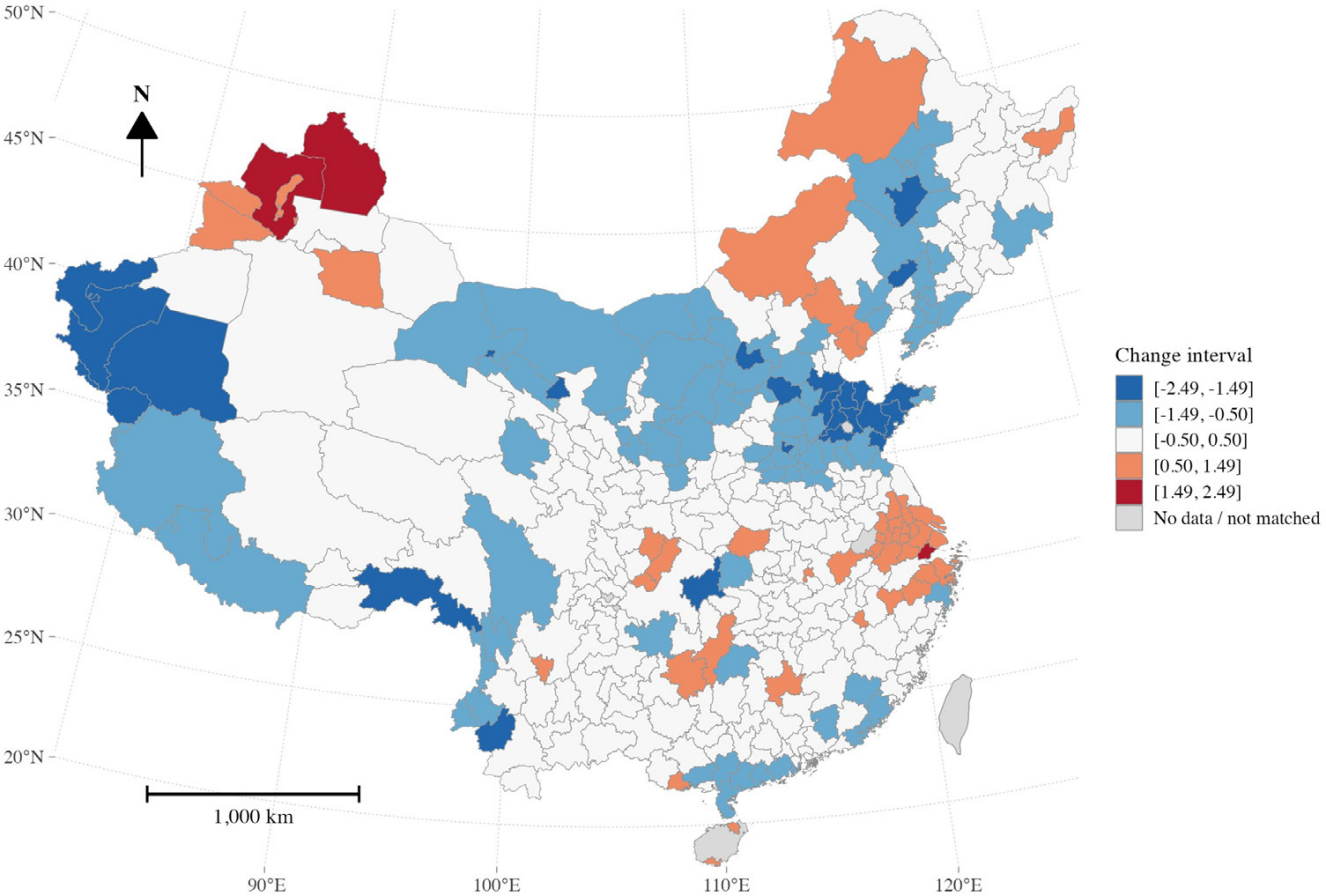
Long-run change in the average city-level composite tail-risk index (TailRisk_city), 2005–2024 minus 1973–1989.

Physical climate risk data are increasingly used in economics, finance, environmental policy, and urban analysis [[1], [2], [3], [4], [5]]. Many existing measures rely on raw weather realizations, disaster records, or threshold counts of extreme events [[1], [2], [3], [4],^6^]. These approaches document important aspects of climate exposure, but they do not always separate abnormal local tail realizations from persistent climatic trends and recurrent seasonal patterns [2,4,6]. The present repository was compiled to provide a city-year panel that measures tail physical climate risk from residual daily weather processes after city-specific preprocessing. The construction starts from harmonized daily precipitation and temperature records for Chinese cities and reduces each city-variable series to a seasonal residual using a low-order Fourier climatology in day of year, with no time trend or calendar fixed effect removed. Annual tail exposure is then accumulated as exceedance intensities over a grid of city-specific quantile supports and standardized into composite precipitation, temperature, and overall city-level tail-risk indices. The released files contain these standardized composite indices, a harmonized city-day weather panel, a city identifier crosswalk, construction code, and supporting documentation. This article is not linked to a separate research article and serves as documentation of the publicly available repository [7] Figs. 1 and 2.

## 3 Data Description

The repository contains the released city-year panel, the harmonized city-day weather panel, a city identifier crosswalk, and three supporting documentation files. Table 1 provides a file-level overview of the repository contents and lists each file by name, format, level, and role in the release. The principal released dataset is the city-year panel, available in Stata and comma-separated formats, in which each observation corresponds to one city in one calendar year. This panel contains the standardized composite tail-risk indices for precipitation, temperature, and the overall city level, administrative identifiers, and geographic information where available Tables 2, 3.

**Table 1:** Overview of the repository contents.

File name	Format	Level	Description
city_year_tailrisk_1973_2024.dta	Stata	City-year	Main released panel containing the standardized composite tail-risk indices (precipitation, temperature, city), city identifiers, and geographic fields
city_year_tailrisk_1973_2024.csv	CSV	City-year	CSV version of the main released city-year panel
city_daily_weather_panel_1973_2024.dta	Stata	City-day	Harmonized daily precipitation and temperature panel used to construct the annual indicators
city_daily_weather_panel_1973_2024.csv	CSV	City-day	CSV version of the harmonized daily weather panel
city_identifier_crosswalk.dta	Stata	City identifier	Standardized city matching file used for harmonization and merging
city_identifier_crosswalk.csv	CSV	City identifier	CSV version of the city identifier crosswalk
variable_dictionary.xlsx	Excel	Documentation	Variable names, definitions, units, and construction notes
construction_code.do	Stata do-file	Code	Main code for preprocessing, seasonal residualization, quantile tail-intensity measurement, and annual index construction
README.md	Markdown	Documentation	Repository structure, file dependencies, and execution order

**Table 2:** Core variables in the city-year panel.

Variable	Description
city_code	Standardized city identifier
city	Harmonized city name
province	Province name
year	Calendar year
TailRisk_precip	Composite precipitation tail physical climate risk index
TailRisk_temp	Composite temperature tail physical climate risk index
TailRisk_city	Composite city-level tail physical climate risk index
lon	Longitude
lat	Latitude
city_type	Administrative category of city unit
valid_days_precip	Number of valid daily precipitation observations in the year
valid_days_temp	Number of valid daily temperature observations in the year

**Table 3:** Summary characteristics of the released dataset.

Characteristic	Value
Time coverage	1973 to 2024
Unique cities	368
Final city-year observations	19,136
Harmonized city-day observations	6981,696
Raw precipitation observations imported	8784,540
Raw temperature observations imported	7019,640
Duplicate city-day observations in final harmonized panel	0
Geographic matching coverage	341 of 368 cities (92.66%)
Annual city coverage	Balanced at 368 cities per year
Missing precipitation in final harmonized panel	0.00%
Missing temperature in final harmonized panel	0.00%

The identifier crosswalk documents standardized city matching used during preprocessing and map linkage. The variable dictionary records variable names, definitions, units, and construction notes. The construction code documents the processing sequence from harmonization to annual aggregation, and the README file describes repository structure and file dependencies.

**Fig. 3:**
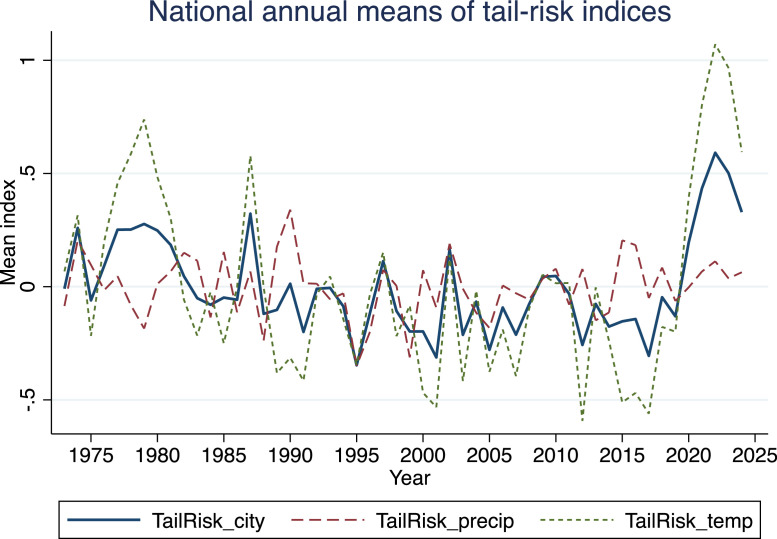
Annual cross-city means of the released composite tail-risk indices (TailRisk_city, TailRisk_precip, TailRisk_temp), 1973–2024.

Fig. 3 reports annual cross-city means of the released composite indices over the full sample period. This repository-oriented presentation follows the structure commonly used in published data articles, where the main text explains the organization of the release and the tables and figures provide the detailed inventory and visual summary of the dataset [[7], [8], [9]].

## 4 Experimental Design, Materials and Methods

### 4.1 Raw data sources

The dataset is constructed from daily land-surface station observations in the Global Historical Climatology Network-Daily archive maintained by the U.S. National Centers for Environmental Information. Four variables enter the construction for each station and day: total precipitation in millimeters and average, maximum, and minimum temperature in degrees Celsius. Each station record is matched to a prefecture-level city under a fixed within-or-nearest-boundary rule, and where a city has more than one qualifying station the daily values are combined into a single city-day series before the records are harmonized into a common city-day weather panel.

### 4.2 Seasonal residuals

Daily precipitation and temperature contain a strong and regular seasonal cycle that is not itself a measure of risk, so each city-variable series is reduced to a seasonal residual before any tail quantity is computed. For city i, variable v, and day d in year t, the residual is the deviation of the observed value from a city-variable seasonal climatology:

(1) $$\varepsilon_{ivdt}=x_{ivdt}-\mu_{iv}\left(s\left(d\right)\right),$$

where x is the observed daily value, μ is the city-variable seasonal climatology, and s(d) is the day-of-year index. The climatology is estimated with a low-order Fourier expansion in day of year, which captures the smooth annual cycle without absorbing short-lived fluctuations:

(2) $$\mu_{iv}\left(s\right)=\alpha_{iv}+\sum_{k=1}^{K}\left[a_{ivk}\cos\left(\gamma_{k}s\right)+b_{ivk}\sin\left(\gamma_{k}s\right)\right],\gamma_{k}=\frac{2\pi k}{365.25}$$

with K set to four annual harmonics and held fixed across cities. Only the seasonal climatology is removed; no time trend or calendar fixed effect is subtracted, so the residual isolates departures from each city's normal seasonal pattern rather than the pattern itself.

### 4.3 City-specific thresholds and tail supports

Extremity is defined relative to each city's own residual distribution rather than by a common absolute cutoff. China spans several climate regimes, from a monsoonal east and south to an arid northwestern interior and the high-altitude Tibetan Plateau, and a fixed meteorological cutoff would label most days extreme in the humid south and almost none in the arid northwest, so the measure would track mean climate rather than tail risk. Let F denote the empirical distribution function of the residuals for city i and variable v. The city-specific quantile threshold at level τ is:

(3) $$q_{iv}\left(\tau \right)=inf\left\{x\in R:{\hat{F}}_{iv}\left(x\right)\geq \tau \right\},$$

For a given threshold, a lower-tail support, an upper-tail support, and the entire-distribution support are defined as:

(4) SivL(τ)={x:x<=iv(τ)},SivU(τ)={x:x>=iv(τ)},SA=R,

The indices are evaluated over a grid of quantile-defined supports: lower-tail supports at τ equal to 0.10, 0.20, 0.30, 0.40, and 0.50; upper-tail supports at τ equal to 0.50, 0.60, 0.70, 0.80, and 0.90; and the entire-distribution support, giving eleven supports per variable. Reporting the full grid avoids selecting a single support after seeing the data.

### 4.4 Annual tail indices

The annual indices accumulate the intensity by which daily residuals exceed the relevant threshold, not the frequency with which they do so. For an upper-tail support the daily exceedance intensity is the amount by which the residual lies above the threshold when it does, and zero otherwise; for a lower-tail support it is the amount by which it lies below:

(5) $$E_{ivdt}^{U}\left(\tau \right)=\left(\varepsilon_{ivdt}-q_{iv}\left(\tau \right)\right)1\left\{\varepsilon_{ivdt}>q_{iv}\left(\tau \right)\right\},E_{ivdt}^{L}\left(\tau \right)=\left(q_{iv}\left(\tau \right)-\varepsilon_{ivdt}\right)1\left\{\varepsilon_{ivdt}<q_{iv}\left(\tau \right)\right\},$$

Averaging the daily exceedance intensity over the valid days in a year gives the annual city-variable tail score:

(6) $$TR_{ivt}\left(\tau \right)=\frac{1}{N_{ivt}}\sum_{d}E_{ivdt}\left(\tau \right),$$

where N is the number of valid daily observations for that city, variable, and year. To form the released indices, the annual tail scores are standardized over the pooled city-year distribution of each variable and aggregated. The temperature index is the average standardized score across the temperature set T equal to {TAVG, TMAX, TMIN}; the precipitation index is the standardized precipitation score; and the city-level composite is their equal-weighted average:

(7) $$\begin{array}{cc} TempTail_{it} & =\frac{1}{\left|T\right|}\sum_{v\in T}z\left(TR_{ivt}\right),PrecTail_{it}=z\left(TR_{i,PRCT,t}\right),CompositeRisk_{it}\\ & \neq 7\frac{1}{2}\left(TempTail_{it}+PrecTail_{it}\right). \end{array}$$

These three standardized indices are released in the city-year panel as TailRisk_temp, TailRisk_precip, and TailRisk_city. They are descriptive summaries of realized exceedance intensity, expressed in common z-score units, so a high value indicates that a city experienced unusually intense exceedances in a given year relative to its own history. The same residuals and supports also support a test for structural change in the residual distribution at an unknown date, developed in the companion study; the released panel reports the annual indices rather than the test output.

### 4.5 Quality control and reuse

Several quality-control screens are applied during construction. Duplicate city-day records are removed, missing-value diagnostics are computed by year and variable, and harmonized city identifiers are checked against the geographic crosswalk. A city-year enters the annual indices for a variable only when its valid daily coverage meets a fixed minimum, and missing days are not imputed. On the common panel these screens yield a balanced set of 18,972 valid daily observations per city, with zero missing precipitation or temperature values and equal observation counts across variables. The resulting city-year panel can be reused in studies of climate-related economic exposure, financial risk, urban vulnerability, and local adaptation, and it can be merged with firm headquarters and subsidiary locations for firm-level applications [[3], [4], [5],^8^].

## Limitations

Several limitations should be noted. First, the quality and continuity of the raw daily weather files vary across cities and years, so users should review variable definitions and documentation when constructing derived measures. Second, the residual-based tail classification depends on the preprocessing specification used to remove trend and seasonality, and alternative specifications may generate different residual series and tail-event assignments [10,11]. Third, the released annual indicators are defined relative to city-specific residual distributions rather than fixed absolute meteorological thresholds. Researchers requiring absolute cutoffs should reconstruct alternative indicators from the harmonized city-day panel. Fourth, geographic matching is high but not complete, and long-horizon administrative naming differences remain an inherent constraint in city-level harmonization. The repository includes a city identifier crosswalk to support verification, extension, and linkage to external sources.

## Ethics Statement

The authors have read and followed the ethical requirements for publication in Data in Brief. This article uses observational environmental data and administrative geographic information. It does not involve human subjects, animal experiments, or any data collected from social media platforms.

## Credit Author Statement

**ZheYu Guo:** Conceptualization, Methodology, Software, Data curation, Formal analysis, Visualization, Writing, original draft. **Han Sun:** Conceptualization, Methodology, Validation, Supervision, Writing, review and editing. **Hyoung-Goo Kang:** Supervision, Validation, Writing, review and editing. **XinYu Zhang:** Data curation, Validation, Writing, review and editing.

## Data Availability

ZenodoA city-year dataset of residual-based tail physical climate risk for Chinese cities, 1973 to 2024 (Original data).

## References

[1] World Meteorological Organization (WMO) (2024).

[2] Rising J., Tedesco M., Piontek F. (2022). The missing risks of climate change. Nature.

[3] Dietz S., Bowen A., Dixon C. (2016). Climate value at risk of global financial assets. Nat. Clim. Change.

[4] Zhang D., Wu Y., Ji Q., Guo K., Lucey B. (2024). Climate impacts on the loan quality of Chinese regional commercial banks. J. Int. Money Finance.

[5] Shala I., Schumacher B. (2024). The impact of natural disasters on banks’ impairment flow: evidence from Germany. J. Clim. Finance.

[6] Lee S.O., Mark N.C., Nauerz J., Rawls J., Wei Z. (2022). Global temperature shocks and real exchange rates. J. Clim. Finance.

[7] Guo K., Ji Q., Zhang D. (2024). A dataset to measure global climate physical risk. Data Br..

[8] Herwig N., Hommel B., Felgentreu D. (2023). Copper distribution in German vineyards and its impact on soil organisms – Dataset of physical, chemical and biological soil parameters of a field survey from 2010 to 2014. Data Br..

[9] Halvorsen C.J., Kelley B., Emerman J., Weiss S., Gleicher D., Stolmeier J., Lush M. (2022). A nationally representative dataset of 1549 Americans aged 18 to 94 on interest in, experience with, and barriers to cogeneration, defined as working with older and younger people for social good. Data Br..

[10] Lu H., Ker A.P. (2024). Testing for distributional structural change with unknown breaks: application to pricing crop insurance contracts. J. R. Stat. Soc..

[11] Lu H., Ker A.P. (2025). On tail structural change in U.S. climate data. Environmetrics.

